# Precision Psychiatry: Data Integration for Personalized Mental Health Care

**DOI:** 10.1192/j.eurpsy.2026.11110

**Published:** 2026-07-31

**Authors:** N. Bouayed Abdelmoula, B. Abdelmoula

**Affiliations:** LR23ES07 Genomics of Signalopathies at the service of Precision Medicine, Medical University of Sfax, Sfax, Tunisia

## Abstract

**Introduction:**

Precision psychiatry aims to go beyond the main diagnostic categories by identifying biologically and clinically homogeneous subgroups of patients. This approach aims to adapt treatments using integrative biomarkers to improve the accuracy of diagnosis, prognosis and therapeutic results.

**Objectives:**

To examine how the integration of various types of data-enriched with metadata (contextual information) and paradata (details on data collection and interaction) can advance precision psychiatry. More specifically, this integration aims to support classifications based on clinical and biological factors and to guide personalized therapeutic strategies through predictive modeling.

**Methods:**

A review of the literature was carried out using key words: Psychiatry, Precision, Classification, Artificial Intelligence, Genomics, Neuroimaging, integration.

**Results:**

Precision psychiatry has facilitated the reclassification of neurodevelopmental and neurodegenerative disorders, with the emergence of predictive models of treatment response (effectiveness of lithium in bipolar disorder) and risk stratification (schizophrenia outcomes). Advances in omic sciences, neuroimaging, digital phenotyping and machine learning allow the synthesis of multimodal data. Predictive models use biological markers, clinical characteristics, environmental exposures and longitudinal patient data to stratify risks and responsiveness to treatment. However, various challenges remain, in particular the need for large transdiagnostic cohorts, multimodal data integration and ethical considerations. Nevertheless, precision psychiatry promises to increase clinical judgment, reduce trial and error in prescribing, and provide personalized interventions based on each patient’s unique biology and environment.

**Conclusions:**

Precision psychiatry represents a transformative paradigm aimed at adapting psychiatric care based on multimodal data, thus improving diagnosis, treatment and long-term mental health outcomes. AI contributes through several key techniques: diagnostic modeling, which uses machine learning to detect neuroimaging, genetic, speech and behavioral patterns for a more precise classification of disorders such as schizophrenia and depression; treatment response prediction, where algorithms predict individual drug or therapy outcomes based on biological data; risk prediction, which monitors digital footprints (e.g. social media, voice, smartphone data) to predict relapse or the onset of psychiatric disorders; digital phenotyping, the collection and real-time analysis of behavioral data via smartphones or wearable devices to track mental health states; and discovery of biomarkers, where AI identifies genetic, neuroimaging or molecular markers associated with psychiatric disorders to support personalized interventions.

**Disclosure of Interest:**

None Declared

